# Systematic Review of Polygenic Risk Scores for Type 1 and Type 2 Diabetes

**DOI:** 10.3390/ijms21051703

**Published:** 2020-03-02

**Authors:** Felipe Padilla-Martínez, Francois Collin, Miroslaw Kwasniewski, Adam Kretowski

**Affiliations:** 1 Centre for Bioinformatics and Data Analysis, Medical University of Bialystok, 15-276 Bialystok, Poland; francois.collin@umb.edu.pl (F.C.); miroslaw.kwasniewski@umb.edu.pl (M.K.); 2 Clinical Research Centre, Medical University of Bialystok, 15-276 Bialystok, Poland; adamkretowski@wp.pl; 3 Department of Endocrinology, Diabetology and Internal Medicine, Medical University of Bialystok, 15-276 Bialystok, Poland

**Keywords:** polygenic risk score, type 1 diabetes, type 2 diabetes, diagnosis, genetics

## Abstract

Recent studies have led to considerable advances in the identification of genetic variants associated with type 1 and type 2 diabetes. An approach for converting genetic data into a predictive measure of disease susceptibility is to add the risk effects of loci into a polygenic risk score. In order to summarize the recent findings, we conducted a systematic review of studies comparing the accuracy of polygenic risk scores developed during the last two decades. We selected 15 risk scores from three databases (Scopus, Web of Science and PubMed) enrolled in this systematic review. We identified three polygenic risk scores that discriminate between type 1 diabetes patients and healthy people, one that discriminate between type 1 and type 2 diabetes, two that discriminate between type 1 and monogenic diabetes and nine polygenic risk scores that discriminate between type 2 diabetes patients and healthy people. Prediction accuracy of polygenic risk scores was assessed by comparing the area under the curve. The actual benefits, potential obstacles and possible solutions for the implementation of polygenic risk scores in clinical practice were also discussed. Develop strategies to establish the clinical validity of polygenic risk scores by creating a framework for the interpretation of findings and their translation into actual evidence, are the way to demonstrate their utility in medical practice.

## 1. Introduction

Diabetes mellitus is a complex and heterogeneous group of chronic metabolic diseases characterized by hyperglycemia, now recognized as one of the most important public health challenges of the 21st century [1]. The World Health Organization [2], estimated that diabetes was the seventh leading cause of death in 2016, being the direct cause of 1.6 million deaths. In 2014, 8.5% of adults of 18 years and older developed diabetes. Diabetes can be treated and its consequences avoided or delayed with diet, physical activity, medication and regular screening and treatment for complications [2].

Diabetes is commonly divided into three subtypes. Type 1 diabetes (T1D) occurs predominantly in people < 30 years old and is generally thought to be precipitated by an immune-associated destruction of insulin-producing pancreatic beta cells, leading to insulin deficiency and requiring exogenous insulin supplement [3]. Type 2 diabetes (T2D) is a progressive metabolic disease characterized by insulin resistance [4] and eventual functional failure of pancreatic beta cells [5,6]. Maturity-onset diabetes of the young (MODY) is a monogenic form of diabetes showing autosomal dominant mode of inheritance. It accounts for 1%–5% of all the diabetic forms of the young and is characterized by anomalous pancreatic beta-cell activity [7,8,9].

Between 2002 and 2012, there was an incidence increase of 1.8% and 4.8% for T1D and T2D among American youths, respectively. Variations in the prevalence of obesity over time may contribute to variations in insulin resistance and to the increasing incidence of T2D [10]. Differences in incidence have been reported in populations from the same ethnic group living in different environments, thereby highlighting the importance of environmental risk factors [11]. Different approaches in the diagnosis area present potential for reducing the mortality and the incidence of cardiovascular complications among patients with T1D and T2D [12]. There is an international epidemic in diabetes with increased prevalence reported globally [13]; as the proportion of diagnosed cases of diabetes increases, a similar increase in the cases of diabetes errors occur [14]. Errors in the primary care of diabetes are misclassification, miscoding and misdiagnosis. A recent study conducted a cross-sectional study in UK, trying to identify cases with potential errors; a high rate of errors was found (57%) compared with previous studies, demonstrating that the prevalence of errors in people with diabetes in primary care is growing [15,16]. The implications of wrong diagnosis, coding or classification affect optimal treatment regimens and cause inappropriate financial and psychological impacts in such patients. Patients with correct diagnosis achieve a significant improvement in their glycemic control [16,17].

The key mission of genomics medicine aims to predict the genetic disease risk on the basis of an individual’s genotype [18]. Identifying those in the population who are at greater risk of disease can result in an improvement in the healthcare sector and lower costs by reducing unnecessary disease concern and by introducing preemptive therapies of lifestyle changes for those at greater risk [19]. The generation of genome-wide variation data has become common for prediction of metabolic diseases [20,21]. Many of the metabolic diseases such as coronary heart disease, atrial fibrillation and T2D [19,22] have well-established risk loci and likely contain many genetic determinants with effects too small to be detected at genome-wide levels of statistical significance [23]. This demonstrates that all common variants across the genome actually explain a much higher proportion of heritability (50% or more) in many complex traits than could be seen based on a small subset of significant single nucleotide polymorphism (SNP) only [24].

The risk of developing T1D or T2D is influenced by the combination of genetic variation in multiple sites across the genome [25,26]. Over the past decades, large-scale genetic studies have described over 400 distinct genetic signals affecting T2D risk [27] and over 50 with influence on T1D predisposition [28]. Genetic testing for T1D risk is not part of routine clinical care. This may in part be due to very modest individual risk effects of non-human leukocyte antigen (HLA) SNPs, historic expense in genotyping HLA alleles and SNPs, lack of available working treatments and a lack of widespread understanding of the complex HLA nomenclature [29]. The increasing prevalence of T2D is one of the greatest challenges in public health [30]. Although obesity is the strongest predictor of T2D, it is also known that heritability of T2D is 26%–69% depending on age of onset, thus motivating the search for genetic predictors for T2D [31,32,33]. An approach to convert genetic data to a predictive measure of disease susceptibility is to add the risk effects of loci into a single genetic risk score (GRS)-polygenic risk score (PRS) [34,35,36].

Prediction accuracy of a PRS is often assessed by measuring the area under the receiver operating characteristic (ROC) curve (AUC). The AUC compares the rates of true positives (sensitivity) and false positives (1—specificity) accounting for the overall performance of predictive models [37]. The use of PRSs could become useful to identify a group of patients at risk; this will offer substantial clinical benefits while preventing growing morbidity and mortality associated with diabetes [38,39,40,41]. Several research groups have developed diabetes PRSs, fitting the scoring models to their study area [19,36,38,39,42,43,44,45,46,47,48,49,50,51]. All of them have used AUC as a predictive parameter to identify the sensitivity and specificity of the outcome of interest. The estimation of T1D and T2D PRSs can be used for diagnosis-support in scientific and clinical environment. Thus, this review aims to identify and compare the most recent studies where a PRS has been established. These may give a lead for researchers to develop an innovative PRS for T1D and T2D and improve existing ones, taking into account variables that have not been used or exploring cutting-edge algorithms.

## 2. Methods

### 2.1. Search Strategies

The databases for the literature search were chosen based on a recommendation of the optimal database combinations [52] and database accessibility in our institution. The three databases chosen for the literature search were Scopus, Web of Science and PubMed. The databases were searched for studies of polygenic risk score for T1D, T2D and monogenic diabetes, published between 2000 and September 2019. The keywords of the queries were “diabetes”, “type 1”, “type 2”, “maturity onset diabetes of the young”, “genetic risk scores”, “polygenic risk scores” and their combinations: “diabetes type 1 polygenic risk score”, “diabetes type 2 polygenic risk score”, “maturity onset diabetes of the young polygenic risk score” and “diabetes genetic risk scores”.

### 2.2. Study Selection

During the screening stage, the exclusion criteria was based on the criteria “the strengthening the reporting of genetic association studies (STREGA): An extension of the strengthening the reporting of observational studies in epidemiology (STROBE)” [53]. From the 22 items mentioned in the article, we took into account the following 12 items that studies must have extensively explained in order to go forward for the eligibility stage: in the introduction section, objectives; in the methods section, study design, setting, participants, variables, quantitative variables and statistical methods; in the results section, participants, descriptive data and main results; and in the discussion section, limitations and interpretation of the results.

### 2.3. Data Collection Processing

The items collected from the full text and Supplementary Information were first author, year of publication, digital object identifier (DOI) when available, ethnicity of study panel, country, data set of study, validation set when available, number of patients and controls, method of sequencing/genotyping, panel of genes used, the number of SNPs used to obtain the PRS, clinical risk factors, the AUC for the clinical risk factors and the AUC for the PRS.

### 2.4. Synthesis of the Results

The AUC of the PRS was taken into account to assess the accuracy and for inter-PRS comparison purpose. The selected AUCs were grouped into three categories based on the diabetes subtypes to discriminate. The first group included T1D PRS comparison. The second group included T2D PRS comparison. The third group included T1D PRS comparison used to discriminate T1D vs. T2D and T1D vs. monogenic diabetes.

## 3. Results

### 3.1. Selected Studies for the Systematic Review

**Figure 1:**
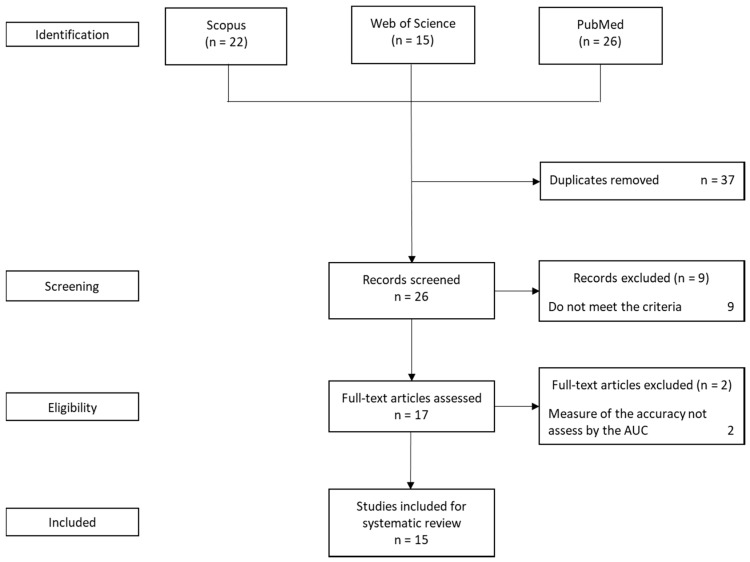
Flow diagram for study selection.

A total of 63 articles were retrieved from Pubmed, Scopus and Web of Science. After removing the duplicates, the total number of studies obtained was 26. The quality of selected studies for the next phase of the screening stage was evaluated using a modified criteria [53], and nine articles were excluded due to the lack of strong arguments on the 12 items of the criteria selected. In the final stage, two articles were excluded as a result of using a different measuring technique for the accuracy of the results. For the systematic review, 15 studies were selected (Figure 1). These studies have varying sources of data sets, panel of genes and genotyping strategies.

There were six studies eligible for the systematic review, which developed PRSs for T1D [38,39,42,43,44,45], and there were nine that studied PRSs for T2D [19,36,46,47,48,49,50,51,54] (Table 1). The majority of the studies were conducted in Caucasian populations, while some of them conducted the studies in Hispanic, African-American, Asian-American, South African and Iranian populations. Apart from the Iranian and South African cohort, all of the other studies had large sample sizes in their patients and their controls. The databases from where the articles were retrieved are shown in Table 1.

The studies relied on datasets from different sources: T1DGC [55], WTCCC [56], UFDI, Iranian Hospitals [35], the PURE study [57], UK hospital [45], GoDARTS [58], MPP [59], BPS [60], Framingham Offspring Study [61], Voight [62], CARDIA [63], the Estonian Biobank [64] and the UK Biobank [65] (Table 2).

The studies also differed in the panel of genes included to obtain the PRS (Table 2). For T1D PRS, the studies combined either the panel of genes from T1DGC (*n* = 4) [55], 1000 genomes project (*n* = 4) [56] or the Immunobase.org on October 2017 (*n* = 1). For T2D PRS, the studies used either specific genes from previous studies (*n* = 6), different versions of the DIAGRAM Consortium panel of genes (*n* = 2) [76] or the 1000 genomes project (*n* =1) [56].

Lastly the studies differed in the platform used for genotyping or sequencing (Table 2). Most of the studies used modified TaqMan assays (*n* = 5), different versions of Affymetrix microarrays (*n* = 5) and Illumina technology (*n* = 4). One study used KASPar genotyping, another the iPLEX technology and another failed reporting the sequencing platform that was operated.

### 3.2. Polygenic Risk Score for T1D prediction

Interacting factors such as background genetic risk, infant and adult diet, environmental exposure, beta-cell stress and immune phenotype increase the development of autoimmunity and beta-cell loss in clinical T1D [29]. Type 1 diabetes has a substantial heritable component, estimated to be between 65% and 88% [77,78]. Genes in the HLA region confer 50% of the genetic risk of T1D. The HLA gene family provides the genetic blueprint for a group of related proteins that help the immune system to discriminate the endogenous proteins from bacterial proteins. The genes in this complex are categorized into two major classes: class I and class II. Class-I HLA presents antigen peptides found within the cell, to CD8 positive (cytotoxic T cells) while Class-II HLA presents antigen peptides found outside the cell, to CD4 positive (helper T cells) [79]. The dominant genetic drivers of this risk are Class II HLA DR and DQ genes on chromosome 6. The HLA haplotypes DR3 and DR4–DQ8, are the two most significant risk haplotypes, with highest genetic risk for T1D occurring in the compound heterozygote [80]. The HLA class 1 alleles have been associated with T1D; A24 is associated with both T1D risk and progression of beta-cell loss [81] and B3906 has been shown to modulate risk when present only with specific class 2 haplotypes [82] and B57 [83]. More than 60 common non-HLA T1D risk variants across the genome have been identified in linkage and genome wide association studies (GWAS) in genes including INS, PTPN22, CTLA-4 and IL2RA [84,85].

Genetic prediction for T1D has evolved from the use of HLA alleles alone [86] to the incorporation of non-HLA variants. In 2014, Winkler et al. developed a multivariate logistic regression model to estimate PRSs including 40 non-HLA genes SNPs, improving significantly the risk score with an AUC of 0.87 comparing to the control [42]. Oram and colleagues [38] adjusted a log-additive PRS model to discriminate patients versus controls for T1D and T2D. They applied a 69 SNP T2D-PRS and a 30 SNP T1D-PRS to a panel of T1D and T2D. They found the T1D-PRS was highly discriminant with an AUC of 0.88, while the T2D-PRS was less discriminant with an AUC of 0.64, and the combination of the two increased slightly the AUC to 0.89. The most recent update for T1D-PRS includes 67 SNPs and accounts for interactions between 18 HLA DR-DQ combinations. This risk score identifies individuals with T1D with an AUC of 0.92 [44] (Table 3).

Currently, the majority of genetics studies on T1D are limited to Caucasian cohorts. However, Perry et al. investigated the hypothesis that race and/or ethnicity would be contextually important for evaluating genetic risk markers previously identified from Caucasian cohorts [43]. They applied the GRS used by Oram [39] to Hispanic Caucasian, African-American and Asian-American populations. The Hispanic Caucasian GRS was highly discriminant with an AUC of 0.90. The GRS for Asian-American was also highly discriminant with an AUC of 0.92, and the analysis indicated that this PRS could discriminate T1D subjects from controls in a small cohort for subjects of Asian-American, but larger studies are required to validate and extend these findings. The African-American obtained a less discriminant GRS with an AUC of 0.75; notable risk differences were observed for three SNPs: SH2B3, CTRB1/2, GAB3 in this population [43] (Table 3).

### 3.3. Polygenic Risk Scores for T2D prediction

The ability of drugs and lifestyle interventions to lead to reduction in progression of diabetes motivates efforts to determine those at the greatest future risk of developing T2D [87,88]. Dozens of genetic loci associated with T2D risk have been discovered using GWAS; currently, 243 have been associated [89,90,91]. There is still some disbelief regarding the practical value of identified SNPs in personalized risk prediction for the disease [92]. The main reason is that the effect of individual SNPs on complex common disease phenotypes is relatively weak; in other words that the environment is the main driver of T2D condition [93].

Before the first GWAS for T2D, a research describing three genetic variants (*KCNJ11*, *PPARG* and *TCF7L2*) that had been associated with T2D was published, assessing the combined risk of these variants and the predictive value of the genetic tests using the AUC. The AUC was 0.58, outpacing the 0.50 value that expressed no discriminative capacity, but short of the values seen for clinical tests [46]. Two years later, Lango and colleagues examined a 16 SNPs PGR; the score, adjusted for age, BMI and sex, predicted diabetes incidence with an AUC of 0.789. Adding the PRS to these clinical factors had only a modest impact on performance, pushing the AUC to 0.80 [47]. In a similar study, a research group assessed 16 SNPs PGR; the score, adjusted for age, sex, family history, BMI, blood pressure, triglycerides and fasting plasma glucose, predicted diabetes incidence with an AUC of 0.740; adding the PRS to clinical risk factors (CRF), the AUC of 0.750 had a small effect on the ability to predict T2D [48]. In the same year Meigs et al. estimated an 18 SNP PRS; the AUC for incident diabetes adjusted for age and sex was 0.534, and an enhanced clinical model incorporating age, sex, family history, BMI, glucose level, cholesterol level and triglyceride level reached 0.90. Adding genetic data to those two PRSs increased the AUC, respectively, to 0.58 and 0.910 [49]. The next analysis was made with a 22 SNP PRS; this group of researchers deduced that a ten-fold increase in effective GWAS sample size for T2D would result in a better performance. The result of the AUC adjusted for age, sex and family history was 0.570; after adding the PRS, the AUC increased to 0.740 [50]. An updated analysis of a 62 SNP PRS generated an improved AUC for T2D prediction. Combined with age and sex, the AUC was 0.72, but after the addition of other important clinical factors the score was 0.91 [51]. A recent study analyzed a South African population; a PRS using only four SNPs was created, taking into account sex, age, BMI and systolic blood pressure as clinical risk factors, generating an AUC of 0.665 [54].

Larger GWAS for T2D have been developed in the last years; these achievements have increased the number of significant loci identified to the hundreds. Although comparing variants that carry out genome-wide significance assures that the variants included in the score represent legitimate associations with disease, an inflexible threshold ignores many other variants, which even if they are truly associated with the phenotype, have escaped detection at genome-wide significance due to defined sample sizes [36]. Khera et al. [19] used a different approach, handling 7 million variants, and after adding sex, age and other important clinical factor, generated an AUC of 0.73 (Table 4).

### 3.4. Polygenic Risk Scores to discriminate different subtypes of diabetes

As it was mention before there are three types of errors in primary care of diabetes, in this section the misclassifications will be reviewed. Misclassification refers to giving the patient a type of diabetes classification that he/she does not have [16]. Diagnosis of diabetes into its subtypes is becoming an increasing dispute. There is a growing problem of obesity in young adults and teenagers, and consequently an increase in T2D; this take us to a misclassification of T1D [94]. There is just a small overlap in the genetics of T1D and T2D, thus a PRS could be used as a diagnostic tool [29,74]. Oram and colleagues [38] were the first ones to accomplish the use of a PRS to discriminate T1D and T2D. They developed a PRS of 30 SNPs, which consists of HLA and non-HLA loci; the AUC was of 0.880, being highly discriminant. They also demonstrated that the use of the top nine SNPs had the same highly discriminant effect that as the use of the 30 SNPs; the AUC for the PRS using the top nine SNPs was 0.873 (Table 5).

Differentiating patients with monogenic diabetes from T1D has important significance in scientific and clinical environments, the treatment in the patient being a crucial factor [95,96]. Patel and colleagues [39], generated a T1D-PRS from 30 SNPs to discriminate monogenic diabetes from T1D. They assessed the ability of the PRS to discriminate T1D and confirmed monogenic diabetes. The analysis of the PRS using the AUC showed that it was highly discriminant, the value being 0.87. The latest study of Yaghootkar and his team [45] provided the first evidence to suggest that the T1D PRS proposed by Oram et al. [38] using the top nine SNPs for a European cohort may help to distinguish monogenic diabetes from T1D in an Iranian population. The AUC analysis showed that the T1D PRS was highly discriminant between monogenic and T1D in the non-European cohort with a value of 0.898, which was similar to the ability of the same PRS in the European cohort (Table 5).

## 4. Discussion

PRSs are powerful tools to support diagnosis; they are consistent throughout life, and thus they could be an effective tool to determine whether a particular patient has T1D, T2D or one of the other forms of diabetes. Thanks to them, it is less difficult to predict the risk of pre-symptomatic diabetes [97]. As stated earlier in the analysis of T1D PRS, with the exception of the cohort of African-Americans [43], the AUC had values of more than 0.80, meaning that all of the PRSs had enough sensitivity and specificity to discriminate patients with T1D. Genetic factors are making an important contribution in the prevention of T1D by giving a reliable risk score. PRS for T1D can diagnose young adults with diabetes that will require insulin treatment in European cohorts, and this will be important to classify accurately patients, when clinical factor make incorrect diagnosis. Using PRS as a tool to discriminate between diabetes subtypes is another advantage provided; the latest studies have shown that T1D PRS is great at discriminating between the patients with T1D versus T2D [38] and monogenic diabetes [39]. The option of using T1D PRS validated for the Caucasian cohort in other ethnicities is under study and could become feasible [45]. The correct diagnosis, as a result of using PRS, could help to generate a lifestyle modification and a pharmaceutical intervention to reduce T1D progression.

Nevertheless, after an extensive review, it was found that there are potential obstacles in the construction of PRSs that could affect how they perform in real world population studies. As it was mentioned in the review, all the studies from the last decade have concluded that clinical risk factors perform quite well in predicting T2D, and there is almost no improvement when adding the PRS; therefore, the PRSs do not have relevant value in the prediction, challenging their clinical relevancy. Further work is still needed to be done to achieve a complete understanding of how PRS is a functional tool for the diagnosis of T2D.

The first obstacle to overcome is the lack of innovation in the generation of PRSs for T2D. Right now, the central target of developing a PRS is to have a correct prediction to recognize individuals at risk [98,99]. The use of SNPs and logistic regression when making a PRS could be improved, since logistic regression is made to understand the process but is not optimized for prediction [100]. There are two approaches to build a PRS model, namely regression-based methods (e.g., logistic regression) and tree-based methods (e.g., random forest) [101,102]. Regression-based methods employ polynomial parametric or non-parametric regression methods to make a relation of the input to the output data. Tree-based methods use the binary split rule to have correlation between the input and output data [103,104,105]. Tree-based methods using machine learning approaches have been extensively used in risk prediction for diseases such as cancer, Alzheimer’s disease, and cardiovascular disease [106,107,108,109]. The use of machine learning techniques, combined with data from GWAS will improve the prediction of polygenic traits [110].

Secondly, the underestimation of population heterogeneity in the prediction of T2D could be the cause of another problem, namely overfitting, which is a common concern in PRS studies. It is required to calibrate, validate and optimize the PRS to every cohort of study to prove that it does not overfit the training data, producing inflated results [18]. The point of reference to avoid the generation of overfit prediction models is to implement a prediction using out of sample data [92,111]. The majority of PRS using Caucasian GWAS are biased by the allelic drift when compared to other populations, even when picking the same variants [25,112]. Diverse ethnic groups have different frequencies of key risk and probably different SNP associations [36]. Therefore, there is a need for developing generalized risk prediction methods and the inclusion of more diverse individuals in risk score studies [25]. To avoid the possibility of obtaining false positive results due to overfitting, the adaptation of existing T2D PRSs validated for Caucasian cohorts to other ethnicities could be the answer.

Thirdly, the environmental effect in genetic studies could be a bias in the development of a T2D PRS, an important aspect to take into consideration. Gene–environment (GxE) interactions can be defined as “genetic effects on a disease that differ in magnitude across environmental contexts” [113]. In most GWAS studies, it is assumed that no GxE interactions exist. If this assumption is incorrect, then the clinical effects of genetic risk factors may be missed [114]. Genetic and environmental factors may jointly contribute to clarify the importance of analyzing GxE interactions [113,114] and the benefits that these interactions have, such as the detection of new disease susceptibility loci [115,116,117]. The high power of PRS approaches to identify GxE interactions has been confirmed [118,119].

A genetic background seems to be fundamental for the development of diabetes, but it is only absolutely enough in Mendelian forms of diabetes, such as monogenic diabetes [120]. The identification of genetic variants using GWAS explain only about 10% of T2D heritability. Studies have assessed the importance of heredity and environment on the etiology of T2D; therefore, the missing heritability may be attributed to GxE interaction [34,121]. Documentation that environmental factors adjust phenotypic expression in genetic risk cohorts has been demonstrated in individuals with glucose intolerance using a multiethnic cohort [87,120], among others studies [122]. Because GxE interactions have proposed as a way to improve genetic risk studies, these environmental factors are relevant to the diagnosis of T2D [123]. The interactions of GxE contribute to the total genetic variance of a given trait [124], demonstrating the importance of GxE interactions in explaining the variance of diabetes-related studies [125]. To discard a bias of the environment in a genetic study, it will be necessary to obtain a similar PRS for T2D prediction when fitting the model on a single homogeneous population but exposed to different environments. In cases where the PRS will be different for the same case exposed before, the neglect of the environment effect will be the reason.

## 5. Conclusions

We have identified 15 studies that developed PRS, 12 to discriminate between patients and controls, and three to discriminate between T1D and diabetes subtypes. We consistently assessed the accuracy of PRSs using the AUC, regardless of the source of data, panel of genes used and genotyping strategies. However, these findings should be taken with caution. These PRSs were identified from 15 studies with variable study designs. In order to have a better prediction of diabetes, the use of PRSs that combine clinical, environmental and genetic interactions must be used. It is necessary to develop strategies to establish the clinical validity of PRSs by creating a pipeline for the interpretation of findings and their translation into actual evidence. Taking into account all the factors for implementation is the way to demonstrate the utility of PRSs in medical practice.

## Figures and Tables

**Table 1:** The studies selected for the systematic review.

Study	Year	Country/Ethnicity	Patients		Controls	Database
**Studies focusing on type 1 diabetes**						
Winkler et al. [42]	2014	Caucasian	4574	1207		PubMed, Scopus
Oram et al. [38]	2015	Caucasian	*n* = 1938			PubMed
Patel et al. [39]	2016	Caucasian	1963	805		PubMed, Scopus
Perry et al. [43]	2018	Caucasian, Hispanic, African-American and Asian-American	627	423		PubMed, Scopus, Web of Science
Sharp et al. [44]	2019	Caucasian	6670	9416		PubMed
Yaghootkar et al. [45]	2019	Iranian	121	6		PubMed, Web of Science
**Studies focusing on type 2 diabetes**						
Weedon et al. [46]	2006	British	2409	3669		PubMed, Scopus
Lango et al. [47]	2008	Scotland	2309	2598		PubMed
Lyssenko et al. [48]	2008	Finland	2201	16,630		PubMed
Meigs et al. [49]	2008	European ancestry in USA	*n* = 2776			PubMed, Scopus, Web of Science
Chatterjee et al. [50]	2013	Caucasian	130	38,987		PubMed
Vassy et al. [51]	2014	European ancestry in USA	5941	5942.		PubMed, Scopus
Läll et al. [36]	2016	Estonia	1181	9092		PubMed, Scopus
Chikowore et al. [54]	2016	South African	178	178		PubMed
Amit et al. [19]	2018	British	26,676	120,280		PubMed, Web of Science

**Table 2:** Data set source, panel of genes used and genotyping strategies.

Study	Year	Data Set	Panel of Genes	Platform
**Studies focusing on type 1 diabetes**				
Winkler et al. [42]	2014	T1DGC	T1DGC	TaqMan 5’nuclease assay
Oram et al. [38]	2015	WTCCC	1000 genomes and T1DGC	Affymetrix 500K SNP chip
Patel et al. [39]	2016	WTCCC	1000 genomes and T1DGC	Affymetrix 500K SNP chip
Perry et al. [43]	2018	University of Florida diabetes institute (UFDI)	Immunobase.org October 2017	Taqman SNP genotyping array
Sharp et al. [44]	2019	T1DGC	1000 genomes	Affymetrix Axiom Array
Yaghootkar et al. [45]	2019	Imam Reza Hospital and Children’s Medical Centre in Iran	1000 genomes and T1DGC	Targeted next- generation sequencing (unspecified)
**Studies focusing on type 2 diabetes**				
Weedon et al. [46]	2006	UK	KCNK11, PPARG, TCF7L2.	Modified TaqMan
Lango et al. [47]	2008	GoDARTS	Frayling [66] and Zeggini et al. [67]	Modified TaqMan
Lyssenko et al. [48]	2008	Malmö Preventive Project (MPP) and Botnia Prospective Study (BPS).	Gloyn et al. [68], Grant et al. [69], Saxena et al. [70], Frayling [66], Scott et al. [71], Sladek et al. [72], Steinthorsdottir et al. [73], Zeggini et al. [74], Zeggini et al. [67], Lyssenko et al. [75].	Allelic discrimination assay-by-design method, Allele-specific (KASPar)
Meigs et al. [49]	2008	The Framingham Offspring Study	Saxena et al. [70], Zeggini et al. [67]	iPLEX technology
Chatterjee et al. [50]	2013	Voight [62]	Voight et al. [62]	Illumina Omni 2.5M Platform
Vassy et al. [51]	2014	The Framingham Offspring Study and CARDIA	DIAGRAMv3	Taqman, Illumina’s OPA technology, Affymetrix 6.0, llumina 370 and 550
Läll et al. [36]	2016	The Estonian Biobank	DIAGRAM Consortium	Illumina Human OmniExpress, Illumina Cardio-MetaboChip
Chikowore et al. [54]	2016	The PURE study	Chikowore et al. [57]	BeadXpress platform, Illumina
Amit et al. [19]	2018	The UK Biobank	1000 genome phase 3 version 5 (Linkage disequilibrium panel)	Affymetrix UK BiLEVE Axiom array, Affymetrix UK Biobank Axiom

**Table 3:** Comparison of the accuracy of T1D PRS assessed by the AUC.

Year	Author	Polygenic Risk Scores	Single-Nucleotide Polymorphism	Area under the Curve for Polygenic Risk Scores	Ethnicity
2014	Winkler et al. [42]	T1D	41	0.87	Caucasian
2015	Oram et al. [38]	T1D	30	0.88	Caucasian
2015	Oram et al. [38]	T1D + T2D	99	0.89	Caucasian
2018	Perry et al. [43]	T1D	32	0.86	Caucasian
2018	Perry et al. [43]	T1D	32	0.90	Caucasian Hispanic
2018	Perry et al. [43]	T1D	32	0.75	African-American
2018	Perry et al. [43]	T1D	32	0.92	Asian-American
2019	Sharp et al. [44]	T1D	67	0.93	Caucasian

**Table 4:** Comparison of the accuracy of T2D PRS assessed by the AUC.

Year	Author	Polygenic Risk Cores (PRS)	Single-Nucleotide Polymorphism	Area under the Curve (AUC) for Clinical Risk Factors	AUC PRS + Clinical Risk Factors	Difference	Clinical Risk Factors	Ethnicity
2006	Weedon et al. [46]	T2D	3	-	0.580	-	-	Caucasian
2008	Lango et al. [47]	T2D	18	0.780	0.800	0.020	Age, BMI, sex	Caucasian
2008	Lyssenko et al. [48]	T2D	16	0.740	0.750	0.010	Age, sex, family, BMI, blood pressure, triglycerides, glucose	Caucasian
2008	Meigs et al. [49]	T2D	18	0.534	0.581	0.047	Age, sex	Caucasian
2008	Meigs et al. [49]	T2D	18	0.595	0.615	0.020	Sex, age, family	Caucasian
2008	Meigs et al. [49]	T2D	18	0.900	0.910	0.010	Age, sex, family, BMI, glucose, cholesterol, triglycerides	Caucasian
2013	Chatterjee et al. [50]	T2D	22	0.570	0.740	0.170	Age, sex, family	Caucasian
2014	Vassy et al. [51]	T2D	62	0.698	0.726	0.028	Age, sex	Caucasian, USA population
2014	Vassy et al. [51]	T2D	62	0.903	0.906	0.003	Sex, family, BMI, blood pressure, HDL cholesterol, triglyceride levels, age	Caucasian, USA population
2016	Läll et al. [36]	T2D-double weighted	1000	0.699	0.74	0.042	Sex, age	Caucasian
2016	Läll et al. [36]	T2D-dw	1000	0.718	0.767	0.049	Sex, age, BMI	Caucasian
2016	Läll et al. [36]	T2D-dw	1000	0.777	0.79	0.012	Sex, age, BMI, hypertension, high blood glucose, physical activity, smoking, food consumption	Caucasian
2016	Chikowore et al. [54]	T2D	4	0.652	0.665	0.013	Sex, age, BMI and blood pressure	African
2018	Amit et al. [19]	T2D	7 million	0.66	0.73	0.070	Sex, age	Caucasian

**Table 5:** Comparison of accuracy of T1D PRS to discriminate diabetes subtypes, assessed by the AUC.

Year	Author	Polygenic Risk Scores	Single-Nucleotide Polymorphism	Area under the Curve for Polygenic Risk Scores	Ethnicity
2015	Oram et al. [38]	T1D vs. T2D	30	0.88	Caucasian
2015	Oram et al. [38]	T1D vs. T2D	9	0.87	Caucasian
2016	Patel et al. [39]	T1D vs. MODY	30	0.87	Caucasian
2019	Yaghootkar et al. [45]	T1D vs. Monogenic	9	0.90	Iranian
